# Safety climate in the surgical center during the Covid-19 pandemic: mixed-method study

**DOI:** 10.1186/s12912-023-01358-x

**Published:** 2023-06-09

**Authors:** Ana Regina Ramos Azevedo, Cintia Silva Fassarella, Daniela Campos de Andrade Lourenção, Flavia Giron Camerini, Danielle de Mendonça Henrique, Renata Flavia Abreu da Silva

**Affiliations:** 1 State University of Rio de Janeiro, Rua Boulevard 28 de Setembro, 157, Vila Isabel. CEP: 20551030, Rio de Janeiro, RJ Brasil; 2 State University of Rio de Janeiro, Rio de Janeiro, RJ Brasil; 3 University of São Paulo, São Paulo, SP Brasil; 4 Federal University of the State of Rio de Janeiro, Rio de Janeiro, RJ Brasil

**Keywords:** Surgicentres, Organisational culture, Nursing, Hospitals teaching, COVID-19

## Abstract

**Context:**

The gradual impact of the Covid-19 pandemic had important effects on routines in surgical environments. In order to cope with the impact and re-establish anaesthesiology and surgery procedures, it was imperative to pursue in-depth studies with a view to ensuring safe surgical care, reducing hazards, as well as protecting the health, safety and wellbeing of the health personnel involved. The purpose of this study was to evaluate quantitative and qualitative approaches to domains of safety climate among multi-professional staffs of surgical centres during the Covid-19 pandemic and to identify intersections.

**Methods:**

This mixed-method project employed a concomitant triangulation strategy on a quantitative approach in an exploratory, descriptive, cross-sectional study, as well as a qualitative approach by way of a descriptive study. Data were collected using the validated, self-applicable Safety Attitudes Questionnaire/Operating Room (SAQ/OR) questionnaire and a semi-structured interview script. The 144 participants were the surgical, anaesthesiology, nursing and support teams working in the surgical centre during the Covid-19 pandemic.

**Results:**

The study found an overall safety climate score of 61.94, the highest-scoring domain being ‘Communication in the surgical environment’ (77.91) and the lowest, ‘Perception of professional performance’ (23.60). On integrating the results, a difference was found between the domains ‘Communication in the surgical environment’ and ‘Working conditions’. However, there was intersection by the ‘Perception of professional performance’ domain, which permeated important categories of the qualitative analysis.

**Conclusions:**

For care practice, it is hoped to encourage improved patient safety, educational interventions to strengthen the patient safety climate and promote in-job wellbeing on the job for health personnel working in surgical centres. It is suggested that further studies explore the subject in greater depth among several surgical centres with mixed methods, so as to permit future comparisons and to monitor the evolving maturity of safety climate.

## Background

Safety climate relates to health care personnel’s perceptions of how the organisation deals with safety issues. For that reason, measuring safety climate offers the opportunity to identify the perceptions and attitudes of multi-professional teams regarding patient safety issues in the surgical environment [1].

As safety climate is intrinsically related to health service quality [2], managers, service providers, professional associations and world and government organisations can be seen to have voiced concern and discussed the maturing of health organisation culture. In that respect, it is proposed that health institutions adopt a non-punitive model of safety climate, which permits open communication and organisational learning [3–5], because such attitudes and behaviour can lead to measures that reduce unnecessary risks associated with health care [6].

The Covid-19 pandemic gradually affected routines in health organisations and surgical environments making it important to assess the safety climate in surgical centres during the pandemic period with a view to planning to address the impact and re-establish anaesthesiology and surgery procedures [2]. Given these challenges, it is imperative to take coordinated, comprehensive measures to prevent and control the Covid-19 pandemix, with a view to health and safety, workforce management and psychosocial support, so as to reduce risks and protect healthcare personnel’s health, safety and well-being. That stance is required because deficient occupational health and safety measures can be an influence in increasing job-related disease rates, high rates of absenteeism, lower productivity and impaired quality of healthcare [7].

Evidence of the pandemic’s impact on safety climate includes postponed surgeries, increased complications for lack of surgical procedures, the risk of Covid-19 transmission during procedures, posing a need for new safety and flow protocols, awareness building as to appropriate, quality, rational use of personal protection equipment, stronger and more effective communication with testing laboratories and routine pre-operatory assessment [8].

Knowing and evaluating safety climate in the perioperatory environment is expected to yield data for a situational diagnosis, continued professional development measures, introduction of care protocols and monitoring of surgical incidents [9]. Furthermore, research allied to strategies for improving work processes can contribute to fostering better quality health services [4].

Data from research into safety climate and culture help guide planning and implementation of measures with a view to creating a satisfactory and propitious working environment, with motivated staff and particularly quality of care and guaranteed safety for patients and surgical team [4].

There is as yet little scientific evidence of safety climate studies that apply an instrument appropriate to the surgical centre setting, making it impossible to compare by the psychometric properties of its domains [10, 9]. Accordingly, of the various questionnaires available for measuring safety climate, this study chose the version translated, adapted and validated for the Brazilian context of the Safety Attitudes Questionnaire/Operating Room (SAQ/OR), because it is specific to the surgical centre [4, 9–11].

In view of the overall adversity resulting from the Covid-19 pandemic, it was decided to resort to a mixed method, which offers the advantage of leveraging the power of sophisticated integration of data from quantitative and qualitative approaches and, in that way, overcoming a series of challenges in the health sciences [12]; in this case, the complexity of investigating safety climate in a hospital context involving a multi-professional surgical team in which each profession has its own particular essence and characteristics. That choice rested on the strong points of both quantitative and qualitative methods, particularly because it minimises the limitations of the two approaches and affords a better understanding of what each approach may achieve when used in isolation [12, 13].

In view of the foregoing, the study hypothesis was that there are intersections between the quantitative and qualitative approaches to evaluating safety climate of a multi-professional surgical centre team during the Covid-19 pandemic. Accordingly, this study examined the quantitative and qualitative approaches of safety climate domains as applied to the multi-professional team of a surgical centre during the Covid-19 pandemic, so as to point up the intersections.

## Method

### Study design

This mixed-method study applied a concomitant triangulation strategy which made it possible to identify assimilations and divergences among the data. The quantitative portion was cross-sectional, exploratory and descriptive, while the qualitative study was descriptive. Data for the two portions were collected simultaneously.

In a more practical context, the mixed method affords a more robust research approach and makes for more in-depth and complete understanding of the problems in health issues, especially in promoting safe care [14, 15]. It also makes it possible to compare between the differing perspectives based on the two different approaches, so as to extrapolate from the datasets and examine intersections between them.

The supports used were: for the quantitative research, Strengthening the Reporting of Observational Studies in Epidemiology (STROBE®) [16]; for the qualitative research, Consolidated Criteria for Reporting Qualitative Research (COREQ): a 32-item checklist for interviews and focus groups [17]; and, so as to provide even greater methodological rigour in the mixed-method study, the Mixed Methods Appraisal Tool (MMAT) [18, 19].

### Study setting

The research setting was a surgical centre at a university hospital of Brazil’s public Unified Health System (Sistema Único de Saúde, SUS) in Rio de Janeiro. This teaching, research and extension facility performs neurosurgery, surgery of the head and neck, ear, nose and throat and in urology, gynaecology, proctology, orthopaedics, vascular and plastic surgery, conventional general surgery and surgery by videolaparoscopy. On average, 324 surgeries are performed a month or approximately 3,888 per year.

### Population and sampling

The study population comprised all staff of the surgical centre. For the quantitative stage based on a population given by a multi-professional team working in the research setting during the pandemic (*N* = 214), sample size was calculated for a simple random sample, to a 95% confidence level and sample loss of 5%. Sample size was thus 137 workers [20] (EPI INFO™ et al.). Sampling was by the convenience technique. The inclusion criteria were: personnel allocated to and working in the surgical centre during the Covid-19 pandemic and interacting with patients directly or indirectly, as recommended by the questionnaire instrument, with at least a 20-h working week in the sector during data collection and more than one month’s experience in the study surgical centre. The exclusion criteria were personnel away from work on holiday or leave.

For the qualitative portion of the study, the inclusion criterion was having taken part in the quantitative stage. Weighted participation by all professional categories working in the surgical centre was ensured. The total number of interviewees was given by data saturation. In the end, 24 staff members took part.

### Data collection instruments

For the quantitative study, the self-administered, SAQ/OR questionnaire was used. That instrument was chosen after a literature review, which revealed the existence of a specific instrument for evaluating surgical centre safety climate. That 40-item instrument is divided into six domains: safety climate, perception of management, perception of stress, working conditions, communication in the surgical environment and perception of job performance [11]. Affirmative responses were scored as “Disagree completely” – 0 points, “Disagree partly” – 25 points, “Neutral” – 50 points, “Agree partly” – 75 points, “Agree completely” – 100 points and “Not applicable”, which generated no score. These scores posteriores were grouped by domain and the score in each was given by calculating the mean of their sum, that is, the items in each domain were added and the result was divided by the number of items in the domain [11].

Scores could range from 0 to 100, with values of 75 or more considered to represent a positive perception of patient safety. Thus 0 represented the worst possible perception and 100, the best.

Qualitative data were collected by semi-structured interview script of six open questions formulated by the researcher from the domains of the SAQ/OR instrument, as follows: ‘How would you rate the safety climate in the surgical environment in the Covid-19 pandemic?’; ‘How do you feel that management deals with safety? Give an example’; ‘How stressed do you feel in your day-to-day work in the Covid-19 pandemic?’; ‘How would you rate working conditions in the Covid-19 pandemic?’; ‘How would you assess communication (information transmission and equipment) in the surgical environment in the Covid-19 pandemic?’; and ‘What do you think of, and how would you rate, your job performance in the Covid-19 pandemic?’.

### Data collection

After approval from the research ethics committee, data were collected in person by a researcher during all shifts from February to May 2021. The staff were invited to take part in the study and, after the aims of the study were explained to them, participants signed a declaration of free and informed consent. Only then were they offered the printed questionnaire. These were collected up and stored in envelopes coded by numerical sequence in which they were returned.

After participants agreed to voice recording in their declaration of free and informed consent, interviews were carried out face-to-face and individually, in the study setting and, for convenience, in the order in which the quantitative questionnaires were returned. The interviews lasted about 15 to 20 min and were recorded digitally. Given the pandemic situation, a distance of 1.5 m was maintained between researcher and participant, and both used masks and visors throughout collection.

During the study, the lead researcher worked in the study setting as a nursing technician and surgical technician. As a result, she already had a relationship with the participants, enabling her to approach them better, explain the study objectives and motivate them with regard to the study. Despite the existing relationship between researcher and setting, ethical requirements and relations with participants were respected in order to ensure data privacy, validity and reliability.

### Data organisation and analysis

The quantitative data were inserted manually into a Microsoft Excel® electronic spreadsheet and then input to R statistics software, version 4.1.0., a freeware tool used to analyse and treat statistical data.

The instrument’s reliability was evaluated by Cronbach alpha. Descriptive statistics were used for analysis of the numerical and categorical variables. In describing staff profiles, the numerical variables (length of experience in the sector, time working in the unit, age and the instrument domains) were displayed in absolute values, means, medians, standard deviations, upper and lower limits. The categorical variables (job position, team, work time format, ethnicity, shift and sex) were displayed as absolute and relative frequencies.

In order to organise the qualitative data, the interviews were transcribed in full and later revised by the lead researcher so that nothing said was suppressed. This was done using the Microsoft Office Word® text editor software, with each interview identified by a number in the order in which they were transcribed and, so as to ensure participant anonymity, they were identified by professional team, viz.: ENF for the nursing team; CIR for surgery; ANEST, anaesthesiology; and AP, auxiliary personnel. The data then underwent content analysis and were interpreted by thematic category.

This stage was carried out using Microsoft Office Excel®, spreadsheets, which permit data analysis. Accordingly, drawing on the theoretical underpinning and the literature findings, the data were analysed and interpreted on a broad view, so as to identify similarities and differences as compared with other studies [21, 22].

The interpreted quantitative and qualitative data were then examined to detect intersections in the findings using the concomitant triangulation strategy to identify convergences and divergences in the data. The mixed method research approach investigates by combining or associating quantitative and qualitative data, integrating the two approaches in the same study and thus permitting the research problem to be understood better [13].

To that end, the findings were presented in tables and charts to elucidate and help understand the information to be analysed. In this way, the scores for each domain were determined, as were the themes in what was said on each domain and the prevalence of mentions in the qualitative approach. That said, the quantitative and qualitative data were analysed separately and then the results from both were compared so that differences and similarities could be highlighted in the data intersection section.

### Ethical considerations

The study was approved by the research ethics committee of the proponent institution under CAAE: 40571920.1.0000.5258 and was conducted in compliance with resolutions current in Brazil. All data were preserved and analysed confidentially and scientifically.

## Results

### Quantitative data

The highest-scoring patient safety climate domain was ‘Communication in the hospital environment’. The study findings demonstrated the reliability of the instrument, which returned an overall Cronbach alpha score of 0.717. The various domains scored as follows: “Safety climate”, 0.784; “Perception of management”, 0.669; “Perception of stress”, 0.757; “Working conditions”, 0.708; “Communication”, 0.616; and “Perception of job performance”, 0.770.

#### Sample characteristics

The quantitative study participants were 144 staff of the university hospital surgical centre. Their social and demographic characteristics were as follows: 73 (50.69%) were male, 75 (52.08%) were under 39 years old. By profession, the team that predominated was medical (surgeons, surgery residents, anaesthesiologists and anaesthesiology residents) with 92 members (63.89%), followed by the nursing team (nurse, chief nurse, surgical technician and circulating nurse and the team of nursing technicians of the post-anaesthesia recovery unit) with 48 (33.33%) and the support team (hygiene, administrative auxiliary and pharmacy technician) with 4 (2.78%). Note that the professional category with most representatives was surgical technicians and circulating nurses, with 38 personnel (26.39%). The participants’ characteristics are shown in Table 1.

**Table 1:** Characteristics of university hospital surgical centre staff. Rio de Janeiro, 2021

Variables	Categories	N	%
Sex	Male	73	50.69
	Female	71	49.31
Age range	< 39 years	75	52.08
	≥ 39 years	63	43.75
	Not informed	6	4.17
Ethnicity	White	93	64.58
	Brown	30	20.83
	Black	19	13.20
	Yellow	1	0.69
	Indigenous	1	0.69
Job position	Surgical technician/Circulating nurse	38	26.39
	Surgeon	32	22.22
	Surgery Resident	28	19.44
	Anaesthesiologist	20	13.89
	Anaesthesiology Resident	12	8.33
	Nurse	5	3.47
	Support team	4	2.78
	Nursing technician (recovery)	4	2.78
	Head Nurse	1	0.69
Team	Medical	92	63.89
	Nursing	48	33.33
	Support	4	2.78

Note: Data from this study (2021)

Source: the author, 2021

#### Overall SAQ/OR safety climate domains

The mean overall SAQ/OR score was 61.94 (dp ± 12.41). Note that, for a patient safety domain to be considered positive required a score of more than 75. The highest-scoring safety climate domain was ‘Communication in the surgical environment’ (77.91) and the lowest-scoring was ‘Perception of job performance’ (23.60). The safety climate domain scores for the study setting are shown in Table 2.

**Table 2:** Domains of safety climate in the surgical centre of a university hospital (*n* = 144). Rio de Janeiro, 2021

Domains	Mean	Standard Deviation
Communication in the surgical environment	77.91	17.17
Safety climate	70.00	18.53
Perception of stress	66.62	25.30
Perception of management	64.07	19.08
Working conditions	63.62	18.59
Perception of job performance	23.60	23.01
Total	61.94	12.41

Note: data from this study (2021)

Source: the author, 2021

### Qualitative data

The qualitative study participants were distributed as follows: 10 members of the nursing team, 7 of anaesthesiology, 6 of surgery and 1 of the support service. It was decided to take the terms that participants used to name units of meaning as the study categories and to discuss the most prevalent.

The categories discussed were as follows: from the ‘Working conditions’ domain (38.99%), ‘COVID-19 Protocol' (18.87%); from the ‘Safety climate’ domain (16.35%), ‘Patient safety’ (8.18%); from the ‘Perception of stress’ domain (13.84%), ‘Feelings’ (8.49%); from the ‘Perception of job performance’ domain (12.58%), ‘Workload’ (4.40%); from the ‘Perception of management’ domain (9.12%), ‘Management attitude’ (4.72%); and, from the ‘Communication in the surgical environment’ domain (9.12%), ‘Communication’ (5.97%). What was said by the study participants is shown in Table 3 separated by domains and categories.

**Table 3:** Participants’ declarations in the qualitative approach. Rio de Janeiro, 2021

Domain	Category			
**Working Conditions**	**Covid-19 Protocol**			
	**Creation of protocols**	[…] we took the Brazilian Association of Surgical Centre, Anaesthesia Recovery and Material and Sterilisation Centre Nurses (*Associação Brasileira de Enfermeiros de Centro Cirúrgico, Recuperação Anestésica e Centro de Material e Esterilização*, SOBECC) as our basis because that was where, as far as I know, the society placed the various recommendations that came out of the Ministry of Health, right, on health surveillance, but like who really guided the OR was our society. So, based on that, we developed a protocol, […]. (ENF-12)	First of all the SOP were developed, which the teams got together and created… That is what was to be followed. (ENF-03)	
**Safety Climate**	**Patient safety**			
	**Commitment to patient safety**	I believe so, yes. From the nurses, medical staff, anaesthesia… Everyone takes responsibility for safety. Always checks the tests, the patient history, tries to locate the correct side for the surgery so as to leave no doubts. So I believe so, yes. (CIR-19)	It was not my perception that safety was reinforced and increased during the pandemic. Although I perceived that to be the intention, but here in our sector I did not have the impression that safety increased. (ANEST-09)	
**Perception of Stress**	**Feelings**			
	**Feelings**	I think that everyone was really lost before, but as I already said, I think that was the general feeling and, until things were settled, everyone was more or less lost, but later they learned from the errors as they went along. (ENF-03)	I worked with more fear. (ANEST-23)	I think people were more concerned with themselves, with their families, but I can understand that. (ENF-12)
**Perception of Job Performance**	**Workload**			
	**Fatigue**	I felt very tired because of the staff reduction in the area. Whoever was left had to work flat out. (ENF-06)	It’s that wear and tear during the shift, because you, in that period, we do a lot of reworking, of redoing things, as regards the protocol, as regards taking over from the team, yes,… multidisciplinary team and having to chase them up, chase them up, go after them to get them to do what’s written, yes… So then, that reworking, that wears you down. (ENF-24)	I even felt more tired when I got out of here, because the shifts were heavier going, more difficult. (ANEST-09)
**Perception of Management**	**Management Attitude**			
	**Lack of information**	Nobody can inform you. You don’t have a figure and we weren’t told: this many patients admitted, this many deaths, this many recovered… I think that’s important. This many staff. […] Information is everything. (CIR-11)	No, that access to feedback was more difficult. Coming up and giving a suggestion or asking why, why it changed, why it happened, that’s more… It doesn’t happen like that, that’s more… That’s not what comes across. All that comes are orders and we obey. There’s nothing like the reason for the changes. (CIR-21)	But I had the impression that maybe the unit leaders should have come to more of the resident people and given more information. (CIR-23)
**Communication in the Surgical Environment**	**Communication**			
	**Meetings as a strategy for horizontal communication**	I think it might be better to have… I don’t know… at some point, Ah, every Friday there will be one session and there we’ll talk about that, talk about how each one is feeling, how the protocol is being done, […] So I think there could be at least some session, at some point in the day. “Ah I’ll set a fixed day and that way there will be one single information”. So there is a certain deficiency in that regard, in receiving communication. (ENF-08)	I think that what is lacking is meetings to like… once a month to say “oh, this happened, we need to improve this”. Otherwise, it seems like everyone is going a different direction and we are left rather not knowing which way to go. (CIR-11)	

*ENF* Nursing, *CIR* Surgical, *ANEST* Anesthesiology

### Data intersection

Having presented the quantitative and qualitative results, the intersections between the approaches will now be shown, indicating the convergences and divergences (Table 4). The first column gives the safety climate domains; the second, the scores in each domain from the quantitative analysis, ordered from highest- to lowest-scoring; the last column shows the most prevalent analytical categories and the percentages indicating the value of each domain in the qualitative analysis.

**Table 4:** Intersection of quantitative and qualitative data. Rio de Janeiro, 2021

SAQ Domains	Quantitative Results	Qualitative Results
Communication in the surgical environment	77.91	Communication among staff, records and surgical equipment (9.12%)
Safety Climate	70.00	Patient safety, patient identification, Covid-19 testing (16.35%)
Perception of management	64.07	Work of management, hospital performance, lack of information (9.12%)
Perception of stress	66.62	Feelings roused, stress, mental health (13,84%)
Working Conditions	63.62	Creation of protocol, difficulty in adherence, nurses as leaders in the surgical centre (38.99%)
Perception of job performance	23.60	Workload, fatigue (12.58%)

Note: data from this study (2021)

Source: the author, 2021

Evaluation of the findings from both approaches revealed divergences: from the quantitative data, the most positive domain for patient safety climate was ‘Communication in the surgical environment’ (77.91) while, from the qualitative data, this domain was the least prevalent in mentions (9.12%).

However, convergence was also found: the themes that made up the ‘Perception of job performance’ domain were present in other categories of the qualitative analysis, such as ‘COVID-19 Protocol’ and ‘Feelings’. This was also the lowest-scoring domain in the quantitative analysis (23.60) and accordingly needs improvement and investment to benefit patient safety. It was mentioned in relation to various categories in the qualitative analysis, however, indicating that the study participants also recognised these needs.

## Discussion

### Intersection of data

From the quantitative data, the domain with the most positive patient safety climate score was ‘Communication in the surgical environment’ and, from the qualitative data, the predominant domain was ‘Working conditions’. There was thus divergence between the findings from statistical analysis and those from the declarations of the multi-professional team in the study setting.

The surgical environment has a particular, complex culture of its own, in which its personnel experience daily stressful situations that can culminate in conflicts in a tense work environment, where relations among different professions can be difficult. In view of this, it is imperative to adopt strategies for negotiation, communication and teamwork with a view to managing possibly conflictual situations [23].

Surgical centre team meetings can be used as a management strategy for getting closer to the realities of the various members of the team, learning their difficulties and thus fostering action focused on strengthening related solutions [24]. Effective dialogue between leaders and teams through such meetings improves the work environment and enables situations experienced by the health care teams to be identified in advance [25].

Management involvement is fundamental to disseminating safety climate and consequently to evaluating, planning and implementing measures for improvement [26]. A climate of trust is necessary in which errors can be considered explicitly and staff can be sure they will not be punished; in that way, there can be learning from adverse events [27]. In order to strengthen the patient safety climate, management should prioritise a model of leadership that is safe, effective, horizontal and participatory and which contemplates the needs of patients and staff [26].

When combating an enemy like Covid-19, unity, collaboration between health teams, good communication and safety measures were extremely necessary. That given, the literature stresses that those measures should be permanent and not temporary, and thus contribute to patient safety [28].

The “Swiss cheese” theory contributes to the feasibility of a safer care environment by encouraging increasingly effective barriers to prevent the metaphorical holes in the cheese from lining up. The idea of this is that, for a more mature safety climate for surgical patients, communication must improve between personnel at all levels of the hierarchy and punitive culture must be abandoned in favour of learning from error [29].

The finding that the ‘Working conditions’ domain was the most prevalent in the qualitative data contributed to characterising the data collection period further: at that time, the health care staffs found themselves terrified by unknowns, learning to deal with situations day after day and, as frontline personnel in the endeavour to combat the Covid-19 pandemic, worried by issues of patient and personal safety [30, 31].

Meanwhile, there was convergence with the ‘Perception of job performance’ domain; although this returned the lowest patient safety climate score (23.60), it was present in some of the qualitative categories. Remembering that this has to do with the impact of fatigue and overwork on job performance, it can be seen that these factors were present in the participants’ declarations in the ‘COVID-19 Protocol’ and ‘Feelings’ categories created for analysis of the qualitative data.

The challenges involved in installing Covid-19 control and prevention protocols caused participants to report fatigue during work; that is, health care personnel recognised fatigue and long working hours to be factors impairing job performance, resulting in loss of efficiency and productivity [32]. A study has indicated that job dissatisfaction among nurses is related to the accumulation of activities and poor prospects of acquiring new knowledge, which can impair the quality of their care for patients [33].

As nursing is considered to form the backbone of health services, more investment must be applied to strategies to identify the psychosocial needs and situations of emotional vulnerability affecting these health care professionals [25]. Fear is among the signs and symptoms of psychological suffering experienced by these professionals, as are anxiety, depression, insomnia and physical and mental exhaustion [25].

The pandemic setting intensified workplace stress and had adverse effects on health care personnel’s quality of life. This may burden the system through illness, absenteeism and leave for health treatment [34]. Miranda [25] noted that the media, news and fake news also contributed and affected health care workers’ mental health.

In view of these findings, it is important that management propose measures for continued professional development of health care teams and modify their perceptions of their importance to care and their fundamental role in patient safety, as well as stimulating a positive safety climate in the institution.

### Quantitative and qualitative metadata

As regards sociodemographic and job characteristics, the male sex predominated (50.69%) and, by profession, the largest team in this study was Medicine (63.89%). Standing against that finding, the nursing team accounted for 78.1% of the participants [35], as in Switzerland (66.6%) [36]. These data are important in order to understand the demographic profile of this study, because these medical specialisations are offered in the study setting and are among the five most numerous specialisations [37].

In the job position category, surgical technicians and circulating nurses were found to predominate (26.39%). In this unit, these are nursing technicians and auxiliaries, as corroborated by other studies [38, 39]. Nursing, which is essential to comprehensive, person-centred care, accounts for the largest proportion of the workforce in health care organisations and has gained strong, decisive recognition in efforts to meet the challenges posed by the international emergency scenario of the Covid-19 pandemic [15]. In view of that, the literature notes that nursing has the power to drive and elevate organisational culture towards a positive, proactive safety climate [40].

The mean overall SAQ/OR score was 61.94, revealing that the staff perceived a neutral safety climate in the work environment. This suggests a considerable lack of initiative to improve these professionals’ perceptions of the safety climate, such as by periodical educational interventions, which are considered of prime importance to improving perceptions of safety climate [4].

It is important to note that patient safety issues have financial, social and psychological impacts by causing, for example, increased health costs and expenditures, emotional distress, pain and temporary or permanent interruption of work, which can affect both patient and institution [26]. A fragile safety climate in the surgical centre can contribute to irreversible events and irreparable harm to patients, health personnel and the institution [41], particularly in times such as the Covid-19 pandemic.

In the pandemic context, health care personnel become preoccupied with workloads, the increasing complexity of the care to be provided and the limited resources available. Accordingly, Covid-19 is expected to have adverse impacts on quality of care and patient safety, making adjustments necessary in health institutions and posing the need to develop evidence-based strategies for reducing possible incidents [42].

In the evaluation of the six domains that make up the SAQ/OR, the findings indicate that only the ‘Communication in the surgical environment’ domain (77.91) was perceived by both groups of professionals as being positive as regards safety attitudes in the work environment, which matches the finding of a study by [4]. It is an important finding, given that this domain is the differential in the instrument specific to this study setting [43].

Communication is of the utmost importance in the surgical setting, so much so that one of the six essential goals for safe surgery is effective communication among the surgical team [44]. In the surgical environment, relations among staff of different professions are intense and close, raising the possibility of an environment of conflictual relations [23]. In that light, what is needed is management that supports communication among staff of all levels in the hierarchy, given that this condition is inversely proportional to the occurrence of patient safety-related errors [45].

The lowest-scoring domain (23.60) was ‘Perception of job performance’, which contributed least to the patient safety climate. Studies in southern Brazil [46] and at a Brazilian university hospital [10] found values of less than 75 in all six domains, suggesting that aspects of the patient safety climate call for investments and improvements.

A current study that examined the impact of Covid-19 on nursing work environments and patient safety culture, considering a period prior to the pandemic and then another after the third critical period of Covid-19, found that most patient safety dimensions were weak and needed to improve. That same study found that only the dimension concerned with teamwork in the units was regarded positively prior to the pandemic and after the third critical period of Covid-19 [42].

Given that the positive responses on patient safety were significantly associated with the quality of the work environment, it is hoped that continuous investment in working conditions and promotion of an open and participatory safety culture will improve the quality of health personnel’s work environment.

In the qualitative analysis, from the ‘working conditions’ domain, which reflects the quality of the work environment, there emerged the category ‘Covid-19 protocol’. This made it possible to learn how the participants regarded the issues with the protocol established to address Covid-19.

Health institutions faced with the pandemic needed to update with a view to improving knowledge of Covid-19 in order to provide appropriate, quality care [47]. In this study, the participants’ interviews confirmed that assertion, because available scientific findings were used to develop frontline protocols for dealing with the Covid-19 pandemic in the institution.

In the ‘Safety climate’ domain, where it is possible to learn staff perceptions of patient safety, the salient category was ‘Patient safety’. The interview participants perceived a commitment to patient safety on the part of the surgical team. All personnel, particularly those occupying positions at the head of the organisation, must make a priority of heightening patient safety and of implementing measures for that purpose, so that positive patient safety results are seen in the work process [26].

In the surgical centre, there is the possibility of infection from patient to staff, but also from staff to patient, considering airway manipulation during anaesthesiology procedures and the difficulties in communication between teams [30]. It is important not only to assure health care for patients, but also so preserve the lives and health of healthcare personnel.

In the ‘Perception of stress’ domain, which considers how staff recognise the influence of stress factors on work routines, the predominant category was ‘Feelings’. Besides the risk of contamination, the Covid-19 pandemic demanded greater attention to the health of health care workers, who commonly display symptoms such as anxiety, depression, loss of sleep quality, fear of being infected or of infecting relatives and also burnout from overwork [34, 48].

Worry about the possibility of contaminating family members is a highly significant psychosocial risk [49]. During this period without precedent in the world, health care personnel have suffered adverse mental health impacts with repercussions in the psychosocial sphere and on their overall wellbeing [48]. The findings in the literature explain what was seen here in the participants’ declarations, which mentioned stress, fear and mental health alterations in dealing with the covid-19 pandemic.

Knowing these conditions helps health institutions identify and develop measures to promote, treat and rehabilitate health personnel psychosocially [25]. It is important that each health care worker individually find coping strategies to promote mental health and reduce stress [34]. A Chinese study identified means by which health workers can cope with stressful situations, including finding psychological material, such as books on mental health, psychological resources in the social media, such as self-help messaging and coping methods and counselling and psychotherapy [50]. Other measures to be considered include a shorter the working day, continued professional development, improved working conditions and in-job social support measures [49].

The ‘Perception of professional performance’ domain addresses the impact of workload and fatigue on professional performance. The category chosen to display the findings was ‘Workload’. Frontline workers combating Covid-19 had their working day modified by overtime and the pace of work [30]. Overtime correlates with job stress and tension [51].

There is a significant correlation between patient safety culture and work environment, burnout, depersonalisation and personal relations; when allied to fatigue, these influence patient safety [52]. From the participants’ declarations, it could be seen how much staff shortages affected perceptions of fatigue during work.

It is indispensable for health care facilities not to make a priority of longer working days for health care personnel, particularly during pandemics [34]. However, the pandemic setting affected the working day, causing alterations from overtime and work pace, while frontline workers in efforts to combat Covid-19 were the most exposed to infection though direct contact with the disease [49].

In outbreaks and pandemics, it is common for health care workers to work longer hours, with no breaks and under heavy pressure, which leads them to fatigue and burnout [53]. Such long working days can lead to care provision errors connected with organisational, environmental and care complexity factors and increase exposure to the infectious agent, leaving workers more exposed to diseases and accidents [49].

The ‘Perception of management’ domain considers staff approval for management attitudes in relation to the patient safety climate. The most prevalent category was ‘Management attitude’. From their declarations, the staff felt safeguarded by the health care facility’s management. In workers’ health care, it is important that personnel feel truly supported and not stigmatised [54]. Attention to workers’ health care needs contributes to mitigating stress, which can strengthen compassion and life satisfaction and reduce job-related burnout and anguish. This results in positive impacts on mental health and on quality of the care provided by these professionals [34].

However, participants reported a lack of information from management, which caused insecurity and uncertainty. Communication is listed in the scientific literature as a strategy for health care personnel to cope with stress while combating Covid-19. This includes leaders’ circulating success stories and constantly updating information on the local situations in the pandemic [25, 55].

Accordingly, it is necessary to invest in quality information on health care workers’ health, so as to measure health indicators appropriately and to guide workplace surveillance and inspection activities to support management decision making, favour health care workplace safety and contribute to health research [49].

Lastly, the ‘Communication in the surgical environment’ domain addresses patient safety-related information shared among health personnel. In this domain, the category ‘Communication’ emerged. The interview participants stated the need for more comprehensive communication using, for example, meetings as a strategy. Surgical centre team meetings can be used as a management strategy for getting closer to the realities of the various members of the team, learning their difficulties and thus fostering action focused on strengthening related solutions [24]. Effective dialogue between leaders and teams through such meetings improves the work environment and enables situations experienced by the health care teams to be identified in advance [25].

It is imperative that staff enjoy relations of trust with management and their peers so that there can be positive communication in all directions. This recommends flexible arrangements that help establish effective channels of communication among different levels of the hierarchy, which can influence error reporting without degrading established relations [56].

The study’s practical contributions to surgical patient care quality and safety during the Covid-19 pandemic are directed to contributing thinking with a view to improvements focused on safe care with quality for surgical patients and, through the findings, to provide a basis for developing educational measures and interventions. Lastly, for science, particularly at a critical time worldwide, the study points to the need to foster research of this kind to encourage a mature, positive patient safety climate to develop and spread in healthcare environments.

#### Limitations of the study

Despite the importance of its findings, the study has limitations, including particularly its being restricted to the surgical centre of one university hospital, the possibility that some interview script question may have induced response bias and the researcher’s relationship with the study setting. Accordingly, it is suggested that further studies be conducted in other institutions in order to validate and generalise the findings.

## Final remarks

From the findings and the intersection between quantitative and qualitative data, the proposed study objective was achieved, in that it was possible to examine the surgical centre safety climate from the perceptions of the multi-professional staff.

These diverged in that, in the quantitative approach, the highest-scoring domain was ‘Communication in the surgical environment’, whereas the participants’ declarations in the qualitative approach indicated ‘Working conditions’. Nonetheless, the data also converged: in the quantitative analysis, the ‘Perception of job performance’ domain revealed the need for improvement measures and also permeated important categories and was often mentioned in the participants’ declarations in the qualitative analysis, thus demonstrating that the health care personnel understood the effects of fatigue and overwork on job activities and the need for investment to modify that situation. That finding was achieved only after examining the intersections between the quantitative and qualitative data.

It is suggested that further studies be conducted worldwide to explore the subject in depth, particularly in a variety of surgical settings and using mixed-method study designs, so as to permit future comparisons and strengthen the patient safety climate. Accordingly, it is hoped that this study can contribute with a view to improving patient safety in the surgical environment, so as to encourage evidence-based educational interventions to strengthen the safety climate and foster more assertive communication among health care personnel working in the surgical centre at all levels of the hierarchy.

## Data Availability

All data generated or analysed during this study are included in this published article and its supplementary information files.
